# Supragingival mycobiome and inter-kingdom interactions in dental caries

**DOI:** 10.1080/20002297.2020.1729305

**Published:** 2020-02-19

**Authors:** Divyashri Baraniya, Tsute Chen, Anubhav Nahar, Fadhl Alakwaa, Jennifer Hill, Marisol Tellez, Amid Ismail, Sumant Puri, Nezar Noor Al-Hebshi

**Affiliations:** a Oral Microbiome Research Laboratory, Department of Oral Health Sciences, Maurice H. Kornberg School of Dentistry, Temple University, Philadelphia, PA, USA; b Department of Microbiology, Forsyth Institute, Cambridge, MA, USA; c Department of Computational Medicine and Bioinformatics, University Michigan, Ann Arbor, MI, USA; d Department of Pediatric Dentistry, Maurice H. Kornberg School of Dentistry, Temple University, Philadelphia, PA, USA; e Department of Oral Health Sciences, Maurice H. Kornberg School of Dentistry, Temple University, Philadelphia, PA, USA

**Keywords:** Bacteria, dental caries, dental plaque, high-Throughput Nucleotide Sequencing, mycobiome

## Abstract

**Background**: Recent studies have reveled the presence of a complex fungal community (mycobiome) in the oral cavity. However, the role of oral mycobiome in dental caries and its interaction with caries-associated bacteria is not yet clear.

**Methods**: Whole-mouth supragingival plaque samples from 30 children (6–10 years old) with no caries, early caries, or advanced caries were sequenced for internal transcribed spacer 2 (ITS-2). The mycobiome profiles were correlated with previously published bacteriome counterparts. Interaction among selected fungal and bacterial species was assessed by co-culture or spent media experiments.

**Results**: Fungal load was extremely low. *Candida, Malassezia, Cryptococcus,* and *Trichoderma* spp. were the most prevalent/abundant taxa. Advanced caries was associated with significantly higher fungal load and prevalence/abundance of *Candida albicans. Cryptococcus neoformans* and *Candida sake* were significantly over-abundant in early caries, while *Malassezia globosa* was significantly enriched in caries-free subjects. *C. albicans* correlated with *Streptococcus mutans* and *Scardovia wiggsiae* among other caries-associated bacteria, while *M. globosa* inversely correlated with caries-associated bacteria. *In-vitro, M. globosa* demonstrated inhibitory properties against *S. mutans*.

**Conclusions**: the results substantiate the potential role of the oral mycobiome, primarily *Candida* species, in dental caries. Inter-kingdom correlations and inhibition of *S. mutans* by *M. globosa* are worth further investigation.

## Introduction

The primary role of bacteria within dental plaque in the etiology of dental caries is well established. However, the specifics of this role have been constantly changing and evolving. For decades, the ‘one pathogen, one disease’ paradigm dominated the study of oral infections, which resulted in implicating a handful of species in the etiology of dental caries [1]. A model in which mutans streptococci initiate the cariogenic process and *Lactobacilli* contribute to its progression has prevailed until recently [2]. However, employing molecular approaches over the last two decades has revealed the high diversity of oral microbial communities and complexity of microbial shifts associated with dental caries, considerably expanding the list of taxa that may be involved in dental caries etiology [3]. Focus, therefore, has shifted from specific oral bacteria to studying the entire oral microbiome as an entity of pathogenicity, using cutting edge technologies such as metagenomics, metatranscriptomics, and metaproteomics [1]. Nevertheless, while the oral cavity hosts a variety of microorganisms, studies have almost entirely focused on the bacterial component of the microbiome, the bacteriome. Little work has been done to explore the oral fungal (mycobiome) or viral (virome) communities and their roles in health and disease.

*Candida albicans* is the most common fungus found in the oral cavity. It has been extensively investigated, classically because of its ability to cause oral mucosal infections, and recently due to the increasing evidence implicating it in the etiology of dental caries especially severe early childhood caries (ECC) [4]. A particularly hot area of research has been the study of synergism between *C. albicans* and *Streptococcus mutans*, as an example of inter-kingdom interaction [5]. However, studies employing high throughput, next-generation sequencing technologies have revealed presence of a complex fungal community in the oral cavity with several core taxa [6], some of which, such as *Malassezia*, can outnumber *Candida* [7]. The potential role this fungal community and taxa other than *Candida* play in dental caries and how they interact with bacteria is not known. So far, there has been only one attempt to study the oral mycobiome associated with dental caries. The 2019 study used sequencing of the Internal Transcribed Spacer 2 (ITS2) to analyze supragingival plaque samples from seventeen 7 to 10-year-old Australian children. It identified 46 fungal species, 17 of which were more abundant in children without caries and 3 in children with caries; surprisingly, *Candida* was not among the caries-associated taxa [8].

We have recently used whole metagenome sequencing (WMS) to study the supragingival microbiome in 30 children with various levels of carious severity [9]. In WMS, all fragments of DNA in a sample are sequenced, which allows exploring the different categories of microorganisms present in the sample. However, despite sequencing at an average depth of 21 million reads per sample, fungi were identified in only two samples, most likely because of their extremely low abundance in dental plaque. In these situations, targeted sequencing in which fungal ITS2 is selectively amplified and sequenced would offer a more sensitive alternative. The objective of this study, therefore, was to use ITS2 sequencing to characterize the mycobiome in the same set of samples and correlate it with caries experience and the bacterial profiles obtained in the parent study. We also carried some experiments to assess the interaction between some fungal and bacterial species in vitro.

## Methods

### Subjects, sampling and DNA extraction

Details of study subject recruitment, inclusion and exclusion criteria, sampling and DNA extraction are described in a previous study [9]. In brief, whole-mouth, supragingival plaque samples were collected from 30 children, 6–10 years old, with no caries, early caries or advanced caries (n = 10, each group) recruited at the Pediatric Dentistry Clinic at the Temple University Kornberg School of Dentistry. Caries status was determined using the International Caries Classification and Management System (ICCMS) [10]. The clinical characteristics are summarized in Supplementary Table 1. For DNA extraction, the samples were first digested with Metapolyzyme (Sigma, USA) – a mixture of six enzymes, two of which (lyticase and chitinase) target the fungal cell wall. DNA was then purified using the ZymoBIOMICS Kit (Zymo Research, Germany), that involves beating with a mixture of 0.1 & 0.5 mm beads. The study was conducted in compliance with ethical standards and was approved by the Temple University’s Institutional Review Board (protocol # 24355)

### Fungal load

The fungal load was assessed by quantification of the ribosomal internal transcribed spacer 2 (ITS2) normalized to bacterial 16S rRNA gene (relative quantification) using SYBR Green-based real-time PCR and 2^–ΔCt^ method. For quantification of fungal ITS2, a 20 µl-reaction containing 10 µl Thunderbird Sybr qPCR mix (Toyobo, Japan), 50nmol ROX reference dye, 0.3 µM of each of the primers ITS3-F (5ʹGCATCGATGAAGAACGCAGC-3ʹ) and ITS4-R (5ʹ-TCCTCCGCTTATTRATATGC-3ʹ) [11] and 25 ng of target DNA. Quantification was performed on a Quantstudio^TM^ 3.0 Real-Time PCR system (Applied Biosystems, USA) using the following thermal cycling conditions: Pre-denaturation for 1 min at 95° C, 40 cycles of denaturation at 90°C for 15 s and extension at 60°C for 90 s with data collection at the end of each extension step. For quantification of bacteria, the 16S rRNA gene primers EubF 5ʹ-AAACTCAAAGGAATTGACGGGG-3ʹ and EubR (5ʹ-TTGCGCTCGTTGCGGGACT-3ʹ) [12] were used. The reaction setup and thermal cycling parameters were similar to fungal qPCR except that the extension step was 1 min. Results were expressed in terms of fungal ITS copy/10 million 16S copy.

### Sequencing and data pre-processing

Library preparation and sequencing were performed at the Australian Centre for Ecogenomics as described previously [13]. Briefly, the ITS2 region was amplified using the primers ITS3-F and ITS4-R, linked to Illumina’s specific adapter sequences in standard PCR conditions. The resultant amplicons (~250–590 bp) were purified, indexed in a second PCR step, pooled in equimolar concentrations, and sequenced using 2X300bp paired-end chemistry on a MiSeq Sequencing system (Illumina, USA).

The resultant paired-end sequences were merged using PEAR [14] with the following parameters: minimum and maximum amplicon length of 250 bp and 580 bp, respectively; minimum overlap of 20 bp; and a p-value of 0.001. Primers were trimmed off and the merged reads were quality-filtered using mothur [15] as follows: reads shorter than 210 bp, with ambiguous bases, with homopolymers >8 bases long or/and with a sliding 50-nucleotide Q-score average of ≤35 were filtered out. Chimeras were checked with Uchime [16], using the self-reference approach. Finally, non-fungal sequences were identified and removed using a preliminary Bayesian classification step in mothur (classify.seqs).

### Taxonomy assignment and downstream analysis

The high-quality, non-chimeric merged reads were classified to the species level using a BLASTn-based algorithm described in a previous study [17], modified for use with fungal ITS sequences [13]. Briefly, the sequences were individually searched against a curated subset of UNITE’s database v7.1 (https://unite.ut.ee/repository.php), comprising fungal ITS sequences of all named species (ftp://www.homd.org/publication_data/20170221/), using an alignment coverage of ≥99% and a percent identity of ≥98.5%. Reads were assigned taxonomy of top hits, and in case of ties, multiple-species taxonomy. Reads with no hits were clustered *de novo* into operational taxonomic units (OTUs), OTUs with less than 100 sequences were removed, and the remaining ones were considered as potentially novel species.

Species present in ≤10% of the samples were excluded. Quantitative Insights Into Microbial Ecology (QIIME) [18] was then used for generating taxonomy plots, calculation of alpha diversity indices, distance matrices, and performing principle coordinate analysis (PCoA). Significance of differences in alpha diversity indices between caries groups was calculated using the non-parametric Kruskal–Wallis test. PCoA analysis was based on abundance and binary Jaccard matrices. Differentially abundant taxa between the different groups were identified using linear discriminant analysis effect size (LEfSe) [19].

### Mycobiome-bacteriome correlation, co-culture and spent media experiments

Correlation between core fungal species (those detected in ≥25% of the samples) and the bacterial species identified in the same samples previously [9] was carried out using Spearman’s correlation coefficient in R [20].

Three fungal species, *C. albicans, Malassezia globosa,* and *Cryptococcus neoformans*, found be differentially abundant between the groups (see results below), were tested against each other in co-culture experiments. *C. albicans* strain CAI4 [21] was co-cultured with *M. globosa* (ATCC MYA-4612) on Leeming and Notman agar [22] and with *C. neoformans* (ATCC 32,045) on Sabouraud Dextrose agar [23]. In one experiment, *C. albicans* was allowed to grow first before the other species was inoculated; the other way around in a second experiment.

The spent media from these species were also tested against *S. mutans*. To prepare spent media, each of the fungal strains was grown in the respective broth (see above) at 30°C for 48 h. The cells were then centrifuged at 5000 RPM for 10 min, the supernatant was filtered through 0.2 µ polyethersulfone filter, and used freshly for testing. For initial screening, 10 μl of each spent medium was spotted on a lawn of *S. mutans* grown on BHI agar with 5% Sheep’s blood. For ones that demonstrated activity, growth curve assays were performed in which *S. mutans* (starting at OD_600_ of 0.1 in BHI) was grown in the presence of 10% and 20% spent media at 37°C for 8 h in a Synergy^TM^ HTX multi-mode microplate reader (BioTek, USA).

## Results

### Sequencing and data preprocessing statistics – fungal load

Raw data have been deposited at and are publicly available from Sequence Read Archive (SRA) under project number PRJNA561125. A total of 2,035,675 reads was generated with an average of 67,856 reads per sample. About 75% of the reads were successfully merged, of which only 28% were retained after quality-filtration; the majority of filtered reads at this step were non-specific sequences that lacked primer sequences. Only 92 chimeras were detected while 42% of the remaining reads were found to represent non-fungal sequences, and thus removed. At this stage, three samples (one from each group) were left with less than 300 reads and were thus excluded from further analysis. Eventually, only 252,051 reads remained for downstream analysis in which 93% could be assigned species-level taxonomy (mean of 8,707 ± 17,942 reads per sample). The detailed statistics are provided in Supplementary Dataset 1.

Supplementary Table 2 provides the Ct values of the fungal and bacterial assays for each sample. The median fungal load was 3.7, 4.7, and 9.3 ITS copy/10 million 16S copy in the caries-free, early caries and advanced caries groups, respectively. The difference was statistically significant (Mann–Whitney test) for the advanced caries group vs. the other two combined.

### General mycological profiles

**Figure 1.**
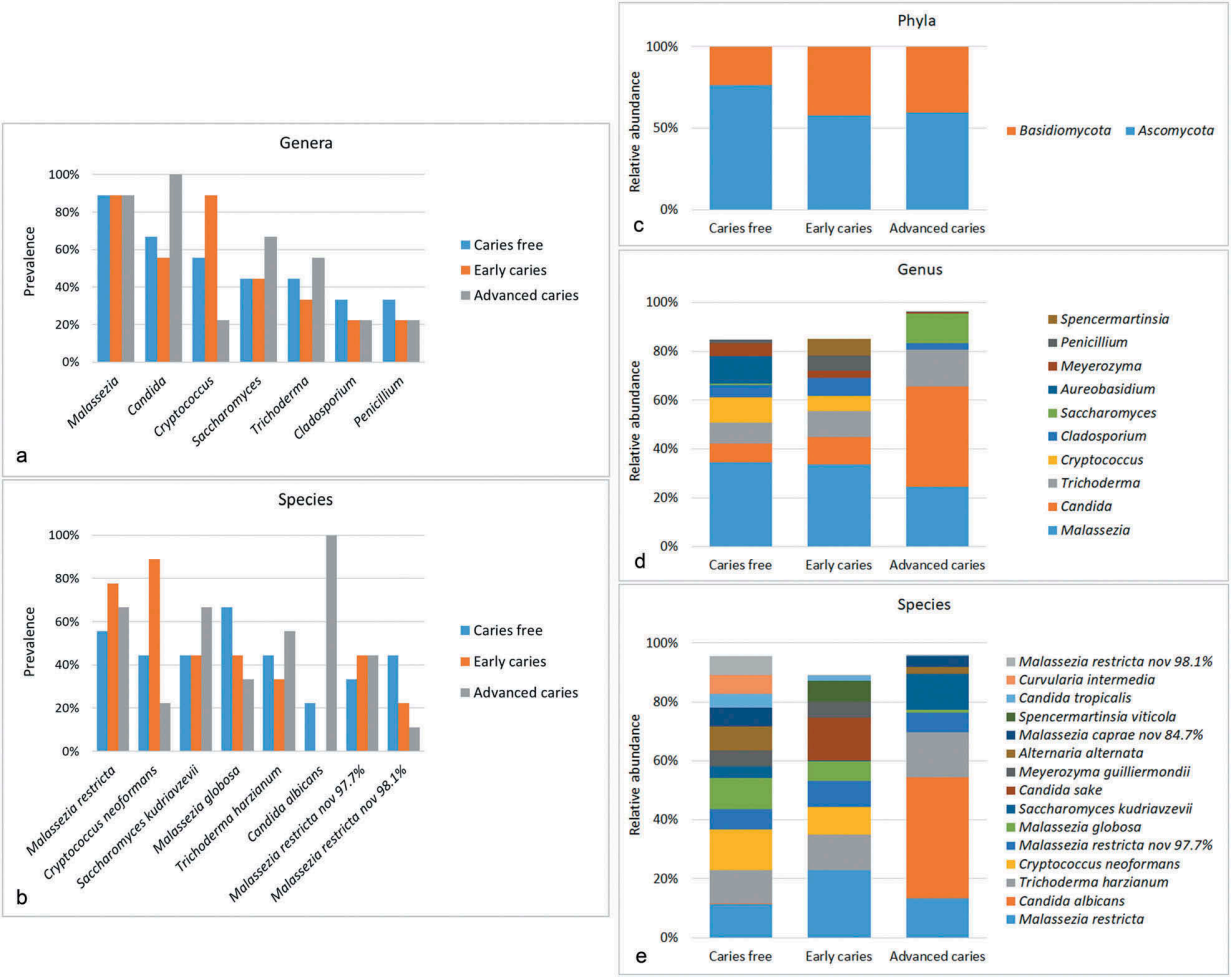
General mycobiome profiles. To the left, detection frequencies of core genera (a) and species (b) (those identified in more than 25% of the samples). To the right, relative abundances of ph1ya (c), top 9 genera (d) and top 15 species (e) identified in the samples (those with average relative abundance ≥2%). For potentially novel species, the name of the closest match and % identity is provided

A total of 102 species and 63 fungal genera belonging to phyla *Basidiomycota* and *Ascomycota* were identified. However, only 23 species and 17 genera were found in ≥10% of the samples and thus included in downstream analysis. The relative abundances and detection frequencies of the excluded taxa are given in Supplementary Dataset 2 and 3. The core genera and species, i.e. those identified in at least 25% of the samples, are presented in Figure 1(a,b). *Malassezia* was the most commonly detected genus followed by *Candida, Trichoderma,* and *Cryptococcus* (detected in ≥50% of the samples). They were also the most abundant in the same order (Figure 1(d)). Together, they constituted nearly 70% of the average mycobiome. At the species level, *Malassezia restricta, C. albicans, Trichoderma harzianum, and C. neoformans* were the most abundant on average and accounted for ~50% of the sequences (Figure 1(e)). However, there were great variations in the relative abundances of taxa across the samples, with some samples dominated by different species, e.g. *Saccharomyces kudriavzevii, Candida tropicalis, Candida Sake*, or *Alternaria alternata* (Supplementary Datasets 4 and 5).

### Diversity and differentially abundant species

**Figure 2.**
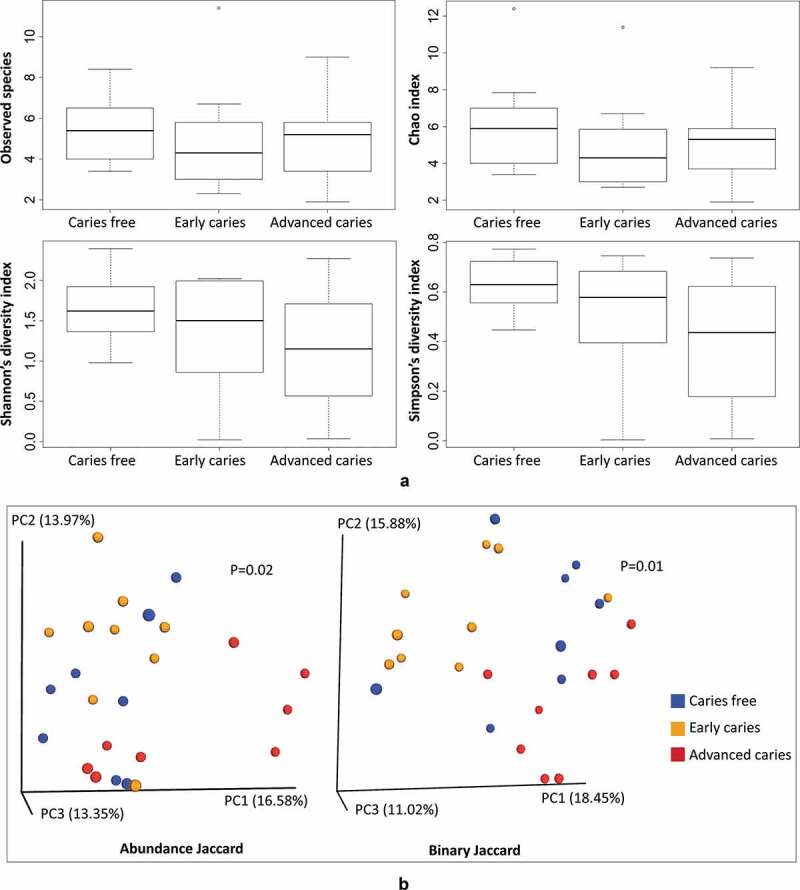
Alpha and beta diversity. (a) A comparison of observed species richness, Chao1 (expected richness), Shannon and Simpson indices between the study groups. (b) PCoA plots of the samples based on abundance Jaccard and binary Jaccard distance matrices. Significance of separation between the groups was assessed with Analysis of Similarities (ANOSIM)

There were no significant differences in species richness and alpha diversity between the study groups as illustrated in Figure 2(a). By PCoA, advanced caries subjects clustered separately from the early caries and caries-free groups; however, there was no distinction between the latter two groups (Figure 2(b)).

**Figure 3.**
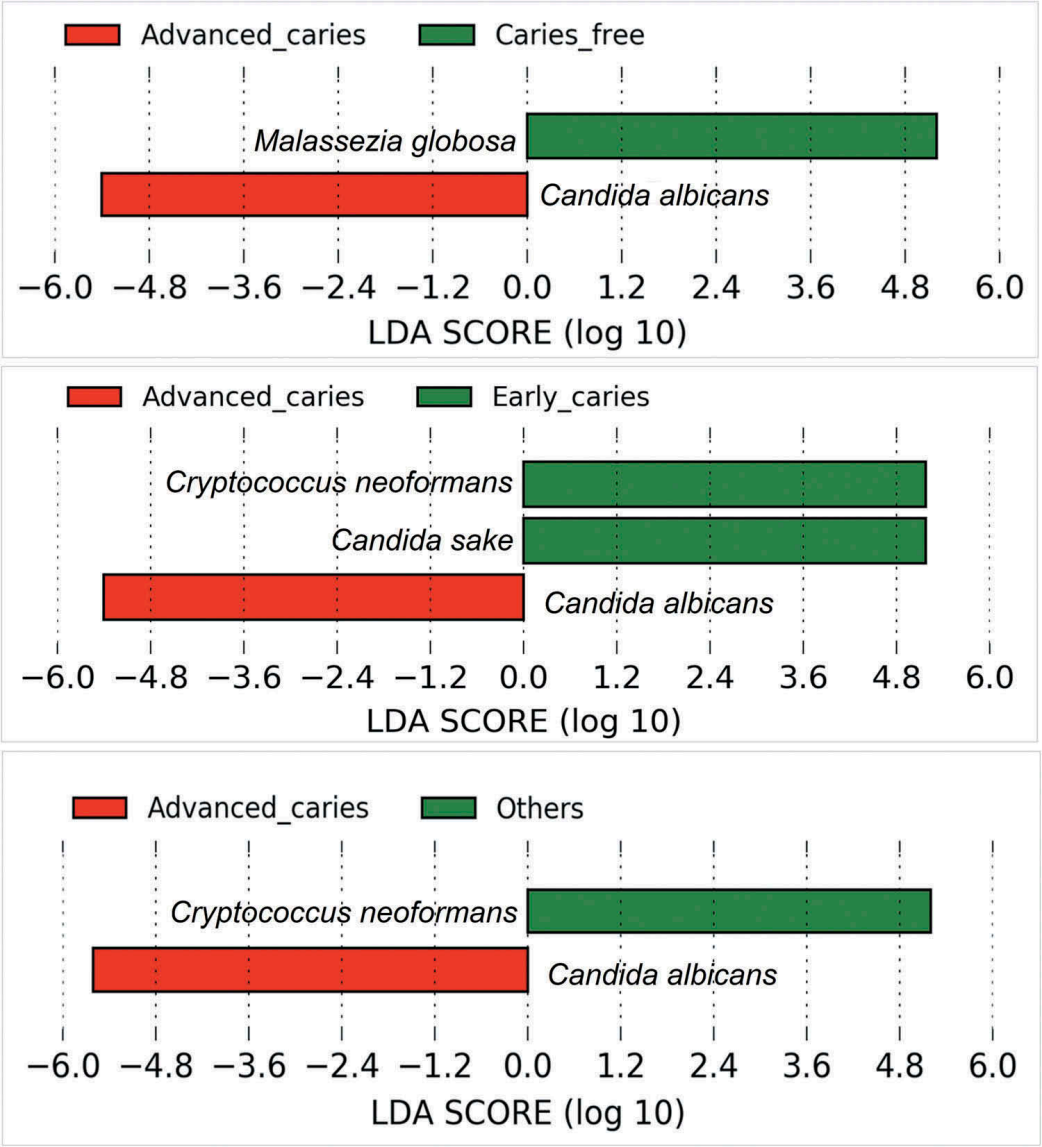
Differentially abundant fungal species. Pairwise comparison between the groups was performed using linear discriminant analysis (LDA) effect size analysis (LEfSe)

**Figure 4.**
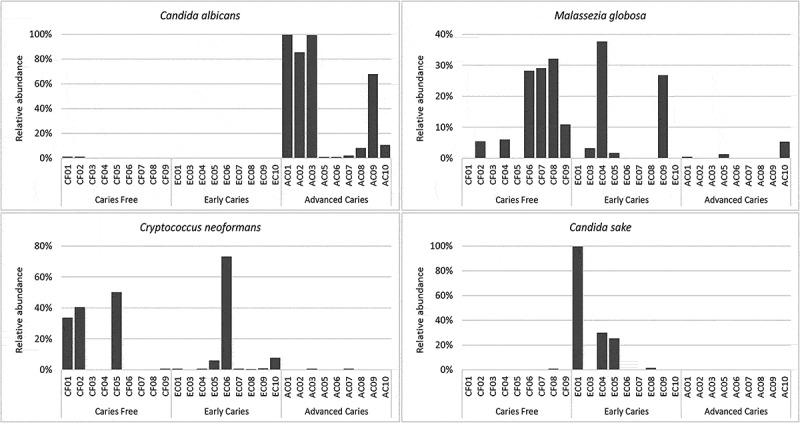
Per sample relative abundances of *C. albicans, M. globosa, C. neoformans,* and *C. sake.*

Differentially abundant species are presented in Figure 3. *Candida albicans* was significantly enriched in the advanced caries group, being detected in all samples (Figure 1(b)). *M. globosa* showed a significant association with the caries-free group while *Candida sake* and *Cryptococcus neoformans* were significantly more abundant in the early caries group. Figure 4 shows the relative abundances of these four species in individual samples.

### Core fungi correlations with bacteria

**Figure 5.**
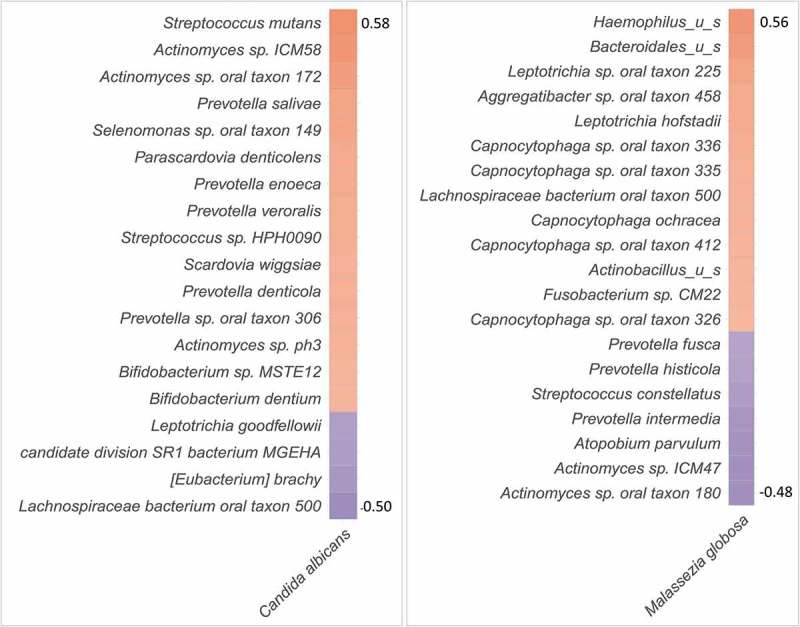
Fungal-bacterial correlation. Correlation matrix for *C. albicans* and *M. globosa* with the bacterial species identified in the same samples in our previous study [9]. Correlations were calculated using Spearman’s coefficient (R Package). Only statistically significant correlations shown (P ≤ 0.05)

Correlations matrices for *C. albicans* and *M. globosa* with bacteria are shown in Figure 5. The strongest correlation for *C. albicans* was with *S. mutans* (r = 0.58). It also positively correlated with species identified in the previous study based on the same samples as caries-associated [9], including *Actinomyces sp. ICM58, Actinomyces sp. oral taxon 172*, and *Prevotella ssp*. In addition, it showed a positive correlation with *Scardovia wiggsiae* and *Bifidobacterium* spp, which are also implicated as cariogens. *M. globosa*, on the other hand, positively correlated with *Leptotrichia* sp. oral taxon 225 which was identified in the previous study as the top health-associated species, while negatively correlated with caries-associated species including *Actinomyces sp. ICM57, Actinomyces sp. oral taxon 180* and *Prevotella* spp. Correlations of the remaining core taxa (*M. restricta, T. harzianum, C. neoformans,* and *S. kudriavzevii)* were less relevant and are presented in Supplementary Figure 1.

### In vitro inter-species interaction

**Figure 6.**
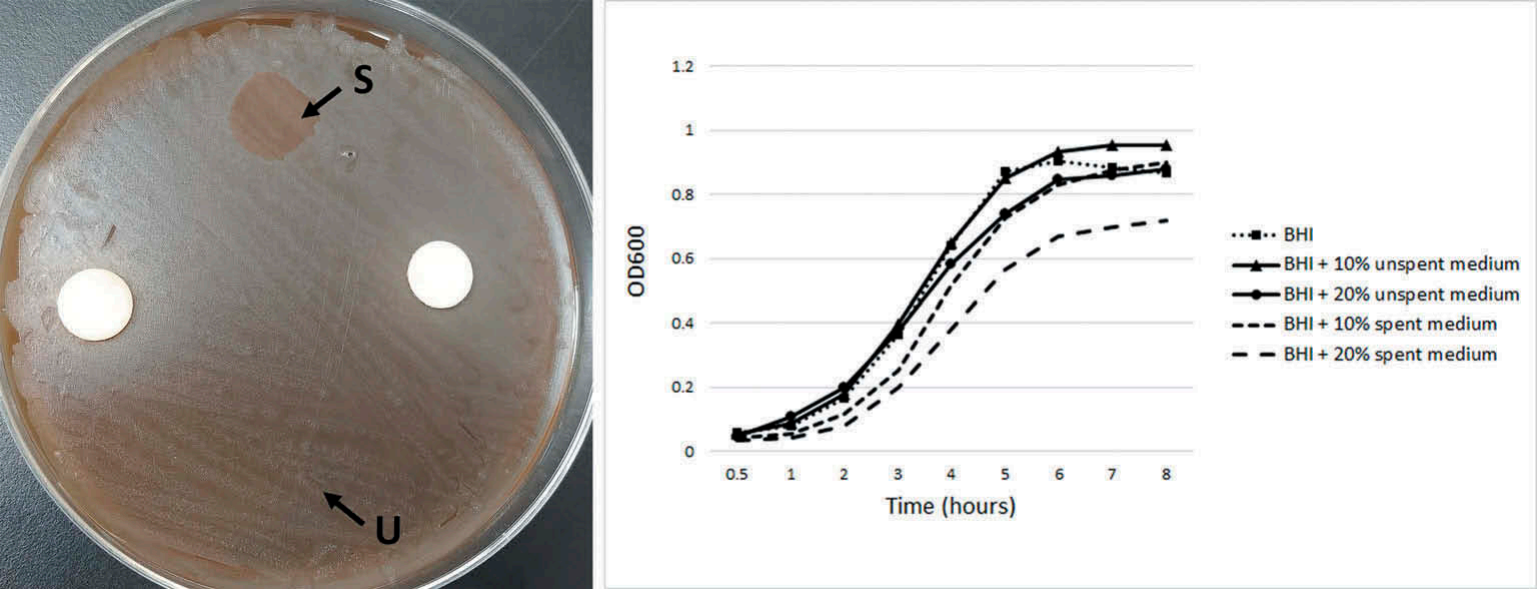
Inhibitory effect of *M. globosa* spent medium on *S. mutans*. To the left, spent media (S) of *M. globosa* was spotted on a lawn of S. mutans. Unspent media (U) was spotted as control. To the right, growth curves of S. mutans in the presence of 10% or 20% spent media of *M. globosa.*

No inhibitory growth effects (contact inhibition or inhibition mediated by secreted active compounds in spent medium) were observed between *C. albicans* and either *M. globosa* or *C. neoformans*, (Supplementary Figure 2). However, spent medium from *M. globosa* resulted in inhibition of *S. mutans* as demonstrated by the spotting as well as the growth curve assays (Figure 6). In the latter, the effect was concentration-dependent, with 20% spent medium causing ~34% reduction in the growth of *S. mutans* compared to ~22% at 10%.

## Discussion

Despite extensive research on the human oral microbiome, little is known about its fungal component, particularly species belonging to genera other than *Candida*. This may have been due to the fact that other fungi are present in very low abundance (rare biosphere), in addition to the difficulty in recovering them using culture-based methods [24,25]. Indeed, in this study we found the fungal load in supragingival plaque to be in the range of 4–10 fungi for each 10 million bacteria (i.e. hardly 0.0001%), which is even lower than that found in saliva (0.06%) [26]. Nevertheless, mycobiome analysis revealed the presence of a complex fungal community, consistent with previous reports [6–8]. Several core taxa identified in this study are in common with those studies, including *Candida, Malassezia, Cryptococcus, Cladosporium*, and *Saccharomyces*, supporting the presence of an endogenous oral mycobiome.

This study is second to that by Fechney et al. (2019) to assess the supragingival mycobiome in association with dental caries. In both studies, the samples were analyzed with ITS2 sequencing. However, apart from identifying *M. globosa* as core species in both, the results from the two studies are largely inconsistent. In the current study, *C. albicans* showed a strong association with dental caries and was almost exclusively identified in children with advanced caries. In contrast, Fechney et al. (2019) found no such association and detected *C. albicans* in all samples from healthy as well as caries subjects. This contradiction may be explained, at least in part, by differences in the selection and grouping of the study subjects between the two studies. While this study included three distinct groups, including a completely caries-free group, the ‘healthy’ group in the Fechney et al. study included a mix of completely caries-free subjects and those with restorations, i.e. with previous caries experience. Making a distinction between early and advanced caries in the current study may also account for differences in the result between the two studies, since the association with *C. albicans* was only observed for the advanced caries group that clustered separately from the other two groups by PCoA. In any case, and apart from this comparison, our findings are in line with growing evidence supporting a potential role of *C. albicans* in dental caries in children [4].

In correlating the fungal profiles to the bacterial counterpart obtained in a previous study [9], *C. albicans* was found to strongly correlate with *S. mutans*, which is consistent with results from culture studies on ECC [27]. In fact, recent *in vitro* and experimental animal work has shown *C. albicans* and *S. mutans* to have synergistic interactions that boost virulence and cariogenicity of biofilms [28,29]. Apart from *S. mutans, C. albicans* also showed a positive correlation with *S. wiggsiae* and *Bifidobacterium spp*, both of which are potential cariogens [30,31]. It also positively correlated with a number of species identified as caries-associated in the previous study. These novel findings suggest extensive interactions between *C. albicans* and caries-associated bacteria other than *S. mutans* that are worth further investigation.

*Malassezia* was the most prevalent and abundant genus, which is similar to the findings reported by Dupuy et al. (2014). This probably explains the increasing interest in studying the role of this genus in oral health and disease. How it survives in the oral cavity in the first place, being a highly lipid-dependent yeast, is an important area of research to explore. This study revealed several novel findings about *M. globosa* in particular. First, *M. globosa* was significantly associated with being caries-free; it also tended to have higher abundance in early caries compared to advanced caries. Second, it correlated positively with health-associated bacteria and negatively with caries-associated bacteria identified in our previous study, indicating extensive inter-kingdom interaction. Indeed, we found the spent medium of *M. globosa* to have an inhibitory effect against *S. mutans*, suggesting it secretes antibacterial substances. *M. globosa* has been recently shown to inhibit biofilm formation by *Staphylococcus aureus* via a secreted aspartyl protease MgSAP1 [32]. Whether this protease or other molecules are responsible for inhibiting *S. mutans* warrants further investigation. In fact, we found the spent medium from *M. globosa* to also inhibit *Streptococcus mitis* (Supplementary Figure 3), which suggests broad-spectrum activity, and calls for the need to explore the interaction of *M. globosa* with a wider range of oral bacteria.

The study has limitations to note. The main limitation is the small sample size, which makes the study underpowered and the results not generalizable. Therefore, the findings should be treated as being preliminary. Another limitation is in connection with the sequencing run: the majority of the reads turned out to represent non-specific or non-fungal sequences, with only 12.4% of the raw reads that ended up remaining for taxonomy assignment. This probably reflects the challenge of amplifying low abundance fungal sequences in very high bacterial DNA background, which requires optimization in future studies. A third limitation is that the in vitro interaction assays were crude and preliminary.

In conclusion, our study identified core fungal species in supragingival plaque that demonstrated differential association with dental caries, and strong correlation and interaction with caries-associated bacteria. The results need to be confirmed in larger-scale studies and further explored in mechanistic in vitro studies.

## Supplementary Material

Supplemental Material
