# Supplementary material for: Supragingival mycobiome and inter-kingdom interactions in dental caries
Source: J Oral Microbiol. 2020 Feb 19;12(1):1729305. doi: 10.1080/20002297.2020.1729305 (PMC7048226; doi:10.1080/20002297.2020.1729305)
Supplement: Supplemental Material [file ZJOM_A_1729305_SM3702.doc]

**Supragingival mycobiome and inter-kingdom interactions in dental caries**

Divyashri Baraniya, Tsute Chen, Anubhav Nahar, Fadhl Alakwaa , Jennifer Hill, Marisol Tellez, Amid Ismail, Sumant Puri, Nezar Noor Al-hebshi

**Supplementary materials**

**Supplementary Table 1. Characteristics of the study subjects**

| **Variable** | **Caries free (n=10)** | **Early caries**  **( n=10)** | **Advanced caries**  **( n=10)** |
| --- | --- | --- | --- |
| **Age** (mean±SD) ^*^ | 8.6±1.07 | 8 ±13.1.63 | 8.2 ±1.4 |
| **Gender:**  Male  Female | 50%  50% | 30%  70% | 40%  60% |
| **Race:**  African American  Caucasian | 80%  20% | 100%  0% | 80%  20% |
| **# Carious lesions** (mean±SD) | 0 | 1.70±2.11 | 2.7±1.05 |
| **DNA yield** (mean±SD) ^*^ | 83.7±33.57 | 144.5±85.1 | 112.4±54.8 |

* Differences insignificant by Kruskal-Wallis test.

**Supplementary Table 2.**

| **Caries group** | **Sample ID** | **Ct - Eubact** | **Ct-fungal** | **2 ^-delta CT^** |
| --- | --- | --- | --- | --- |
| Advanced caries | AC01 | 8.005 | 27.542 | 1.31482E-06 |
| Advanced caries | AC02 | 7.961 | 28.785 | 5.38652E-07 |
| Advanced caries | AC03 | 7.869 | 23.759 | 1.64744E-05 |
| Advanced caries | AC04 | 7.900 | 33.552 | 1.89572E-08 |
| Advanced caries | AC05 | 9.643 | 28.686 | 1.85179E-06 |
| Advanced caries | AC06 | 7.742 | 29.093 | 3.73824E-07 |
| Advanced caries | AC07 | 8.282 | 29.963 | 2.97412E-07 |
| Advanced caries | AC08 | 7.395 | 19.110 | 0.00029744 |
| Advanced caries | AC09 | 8.577 | 26.985 | 2.87507E-06 |
| Advanced caries | AC10 | 7.387 | 28.604 | 4.10317E-07 |
| Early caries | EC01 | 8.076 | 32.934 | 3.28698E-08 |
| Early caries | EC02 | 8.194 | 22.373 | 5.39056E-05 |
| Early caries | EC03 | 7.700 | 31.466 | 7.00818E-08 |
| Early caries | EC04 | 7.631 | 31.784 | 5.36201E-08 |
| Early caries | EC05 | 6.171 | 28.445 | 1.97161E-07 |
| Early caries | EC06 | 9.548 | 28.964 | 1.42954E-06 |
| Early caries | EC07 | 8.157 | 29.896 | 2.85711E-07 |
| Early caries | EC08 | 6.912 | 30.048 | 1.08441E-07 |
| Early caries | EC09 | 6.773 | 30.491 | 7.2475E-08 |
| Early caries | EC10 | 7.190 | 29.860 | 1.49823E-07 |
| Caries free | CF01 | 7.905 | 28.764 | 5.25938E-07 |
| Caries free | CF02 | 8.332 | 24.961 | 9.86856E-06 |
| Caries free | CF03 | 6.854 | 29.309 | 1.73944E-07 |
| Caries free | CF04 | 8.211 | 29.517 | 3.85693E-07 |
| Caries free | CF05 | 6.876 | 29.829 | 1.23105E-07 |
| Caries free | CF06 | 7.298 | 28.745 | 3.49848E-07 |
| Caries free | CF07 | 7.869 | 28.977 | 4.42213E-07 |
| Caries free | CF08 | 7.764 | 28.659 | 5.12651E-07 |
| Caries free | CF09 | 7.242 | 31.181 | 6.21391E-08 |
| Caries free | CF10 | 8.504 | 30.721 | 2.05043E-07 |


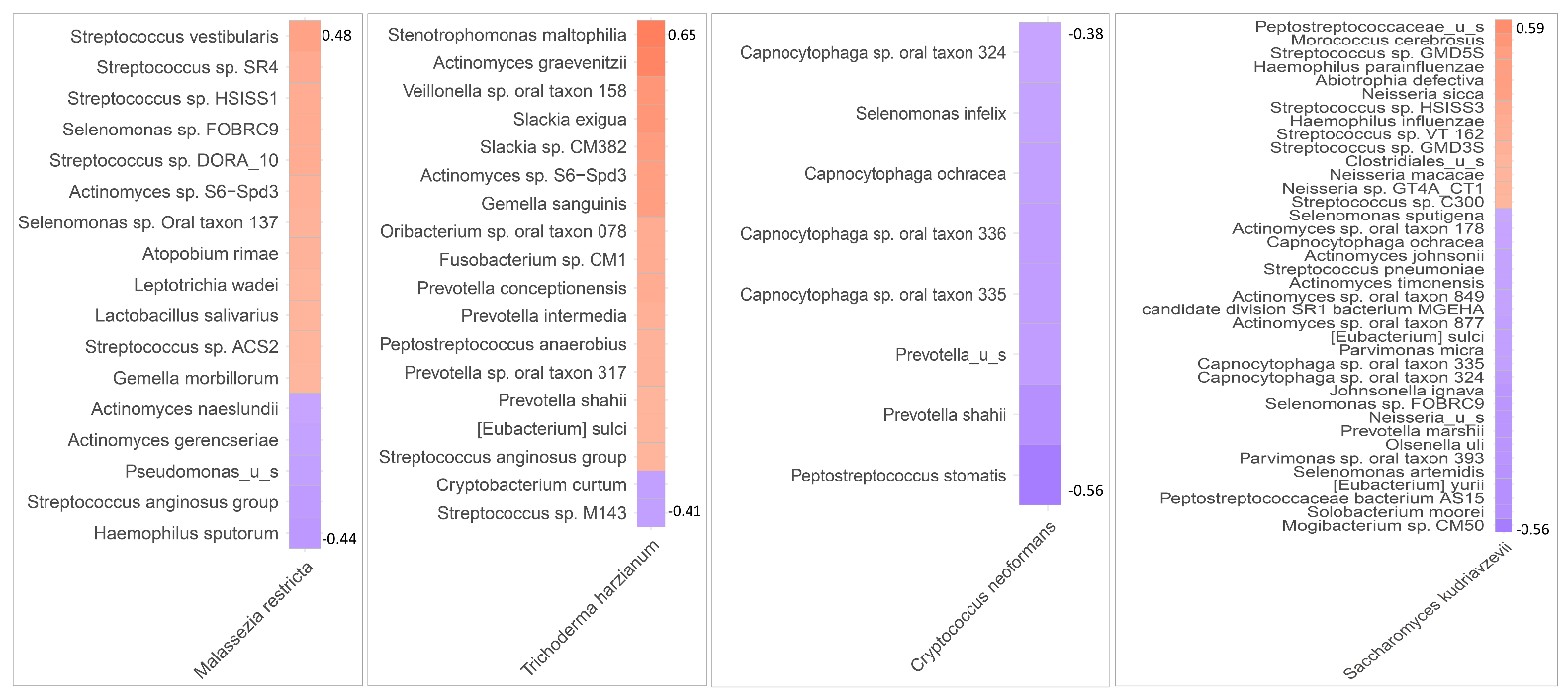
**Supplementary Figure 1.** Correlation of species *Malssezia restricta*, *Trichoderma harzianum*, *Cryptococcus neoformans* and *Saccharomyces kudriavzevii* with the bacterial species identified in the same samples in a previous study. Only statistically significant correlations shown (P≤0.05).

**
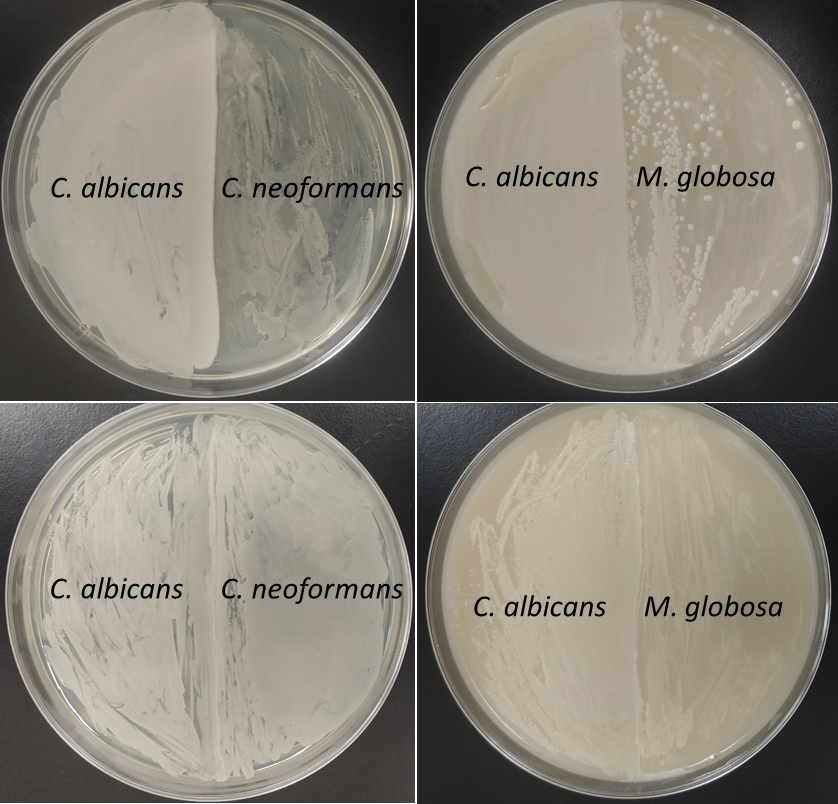
**

//

**Supplementary Figure 2.** **Co-culture experiments.** In the upper panel *Candida albicans* was grown first. In the lower panel *Cryptococcus neoformans* and *Malassezia globosa* were grown first.


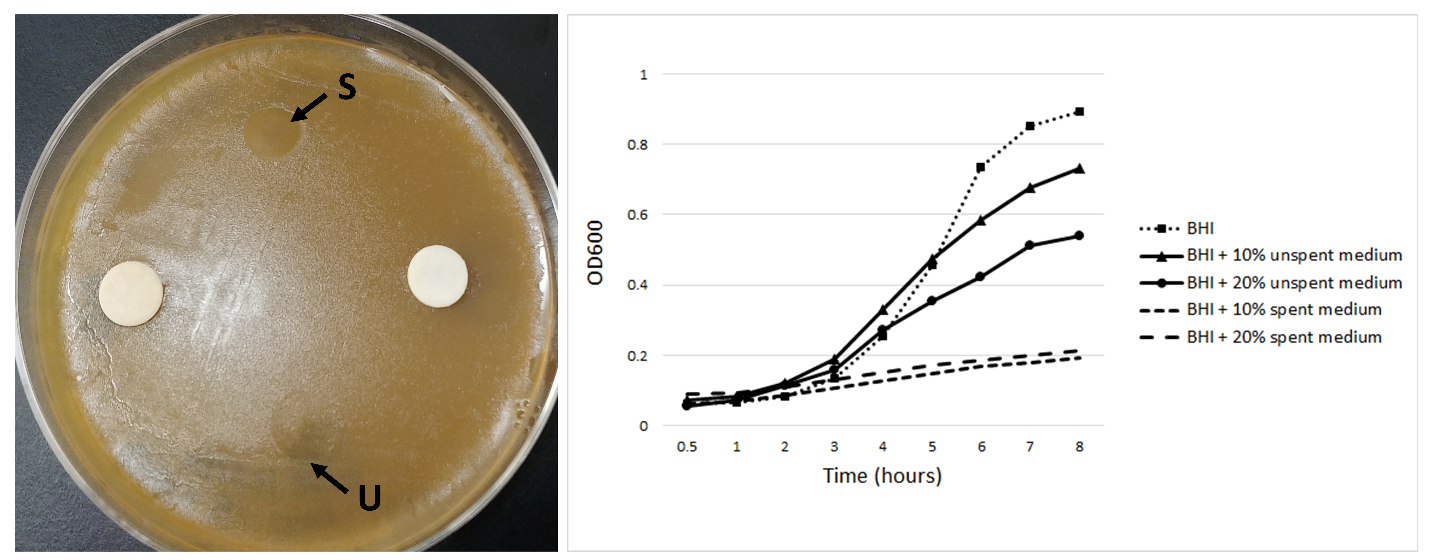


**Supplementary figure 3. Inhibitory effect of *M. globosa* spent medium on *S. mitis*.** To the left, spent media (S) of *M. globosa* was spotted on a lawn of S. mitis. Unspent media (U) was spotted as control. To the right, growth curves of S. mutans in presence of 10% or 20% spent media of *M. globosa.*

**Supplementary Dataset 1.** Reads statistics

[http://www.homd.org/ftp/publication_data/20191015/Sequencing%20statistics.xlsx](http://www.homd.org/ftp/publication_data/20191015/Sequencing statistics.xlsx)

**Supplementary Dataset 2.** Relative abundances and detection frequencies of excluded genera [http://www.homd.org/ftp/publication_data/20191015/Excluded%20genera%20(Detected%20in%20less%20than%2010%25%20of%20samples).xlsx](http://www.homd.org/ftp/publication_data/20191015/Excluded genera (Detected in less than 10%25 of samples).xlsx)

**Supplementary Dataset 3.** Relative abundances and detection frequencies of excluded species [http://www.homd.org/ftp/publication_data/20191015/Excluded%20species%20(Detected%20in%20less%20than%2010%25%20of%20samples).xlsx](http://www.homd.org/ftp/publication_data/20191015/Excluded species (Detected in less than 10%25 of samples).xlsx)

**Supplementary Dataset 4.** Relative abundances and detection frequencies of genera included in the analysis

[http://www.homd.org/ftp/publication_data/20191015/Relative%20abundances%20and%20detection%20frequencies%20-%20Genus%20level.xlsx](http://www.homd.org/ftp/publication_data/20191015/Relative abundances and detection frequencies - Genus level.xlsx)

**Supplementary Dataset 5.** Relative abundances and detection frequencies of species included in the analysis

[http://www.homd.org/ftp/publication_data/20191015/Relative%20abundances%20and%20detection%20frequencies%20-%20species%20level.xlsx](http://www.homd.org/ftp/publication_data/20191015/Relative abundances and detection frequencies - species level.xlsx)

**
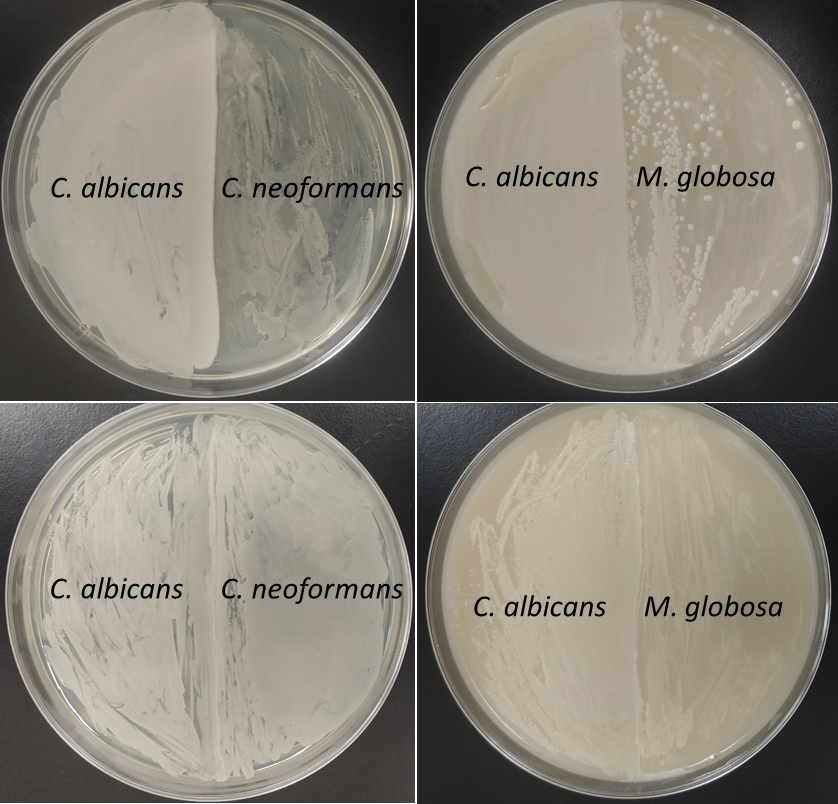

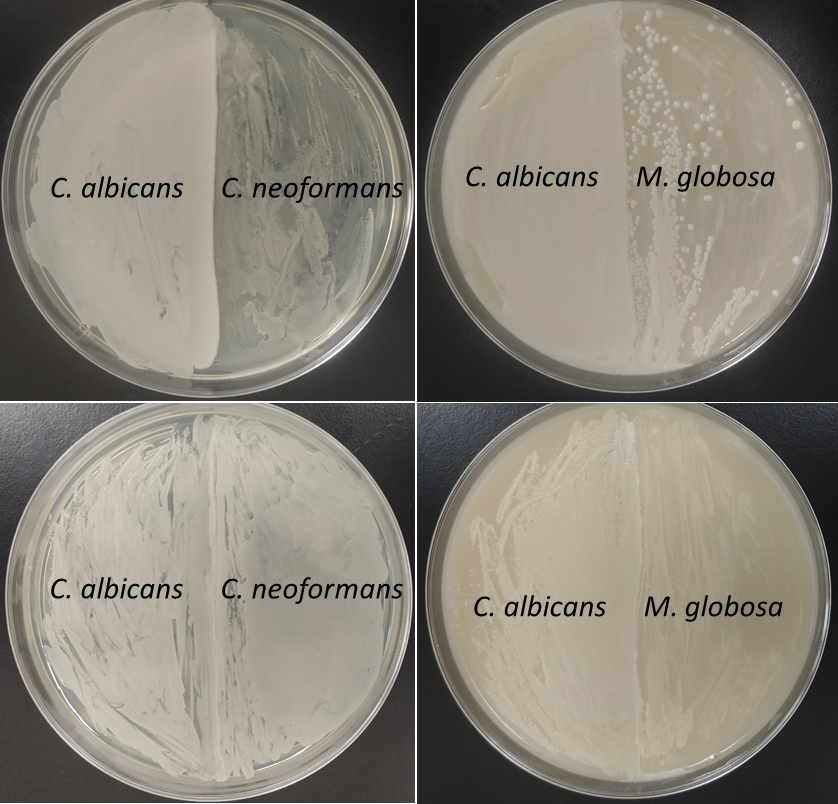

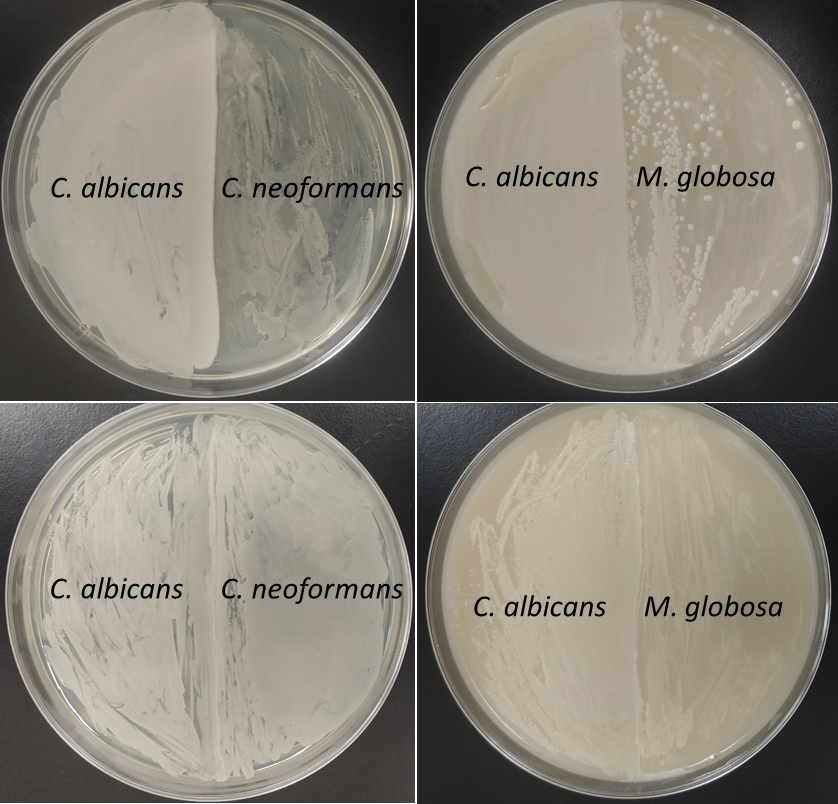

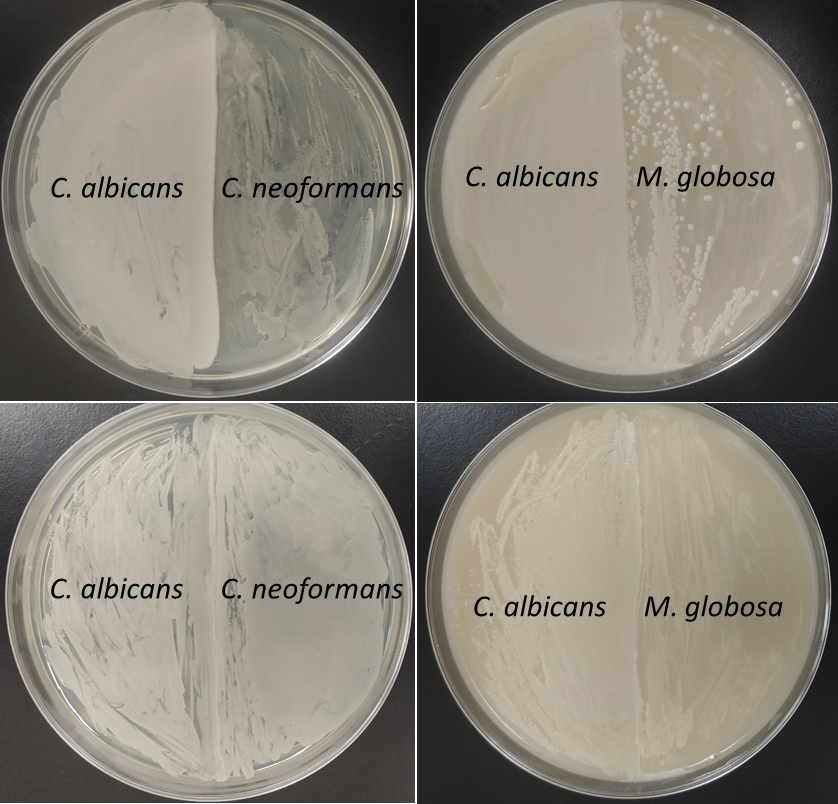

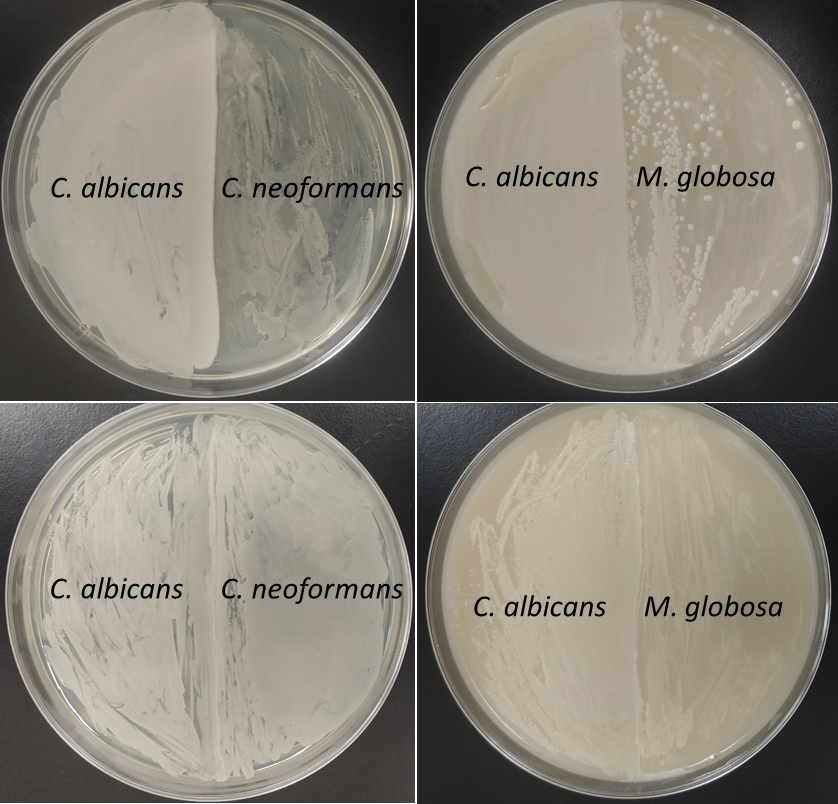

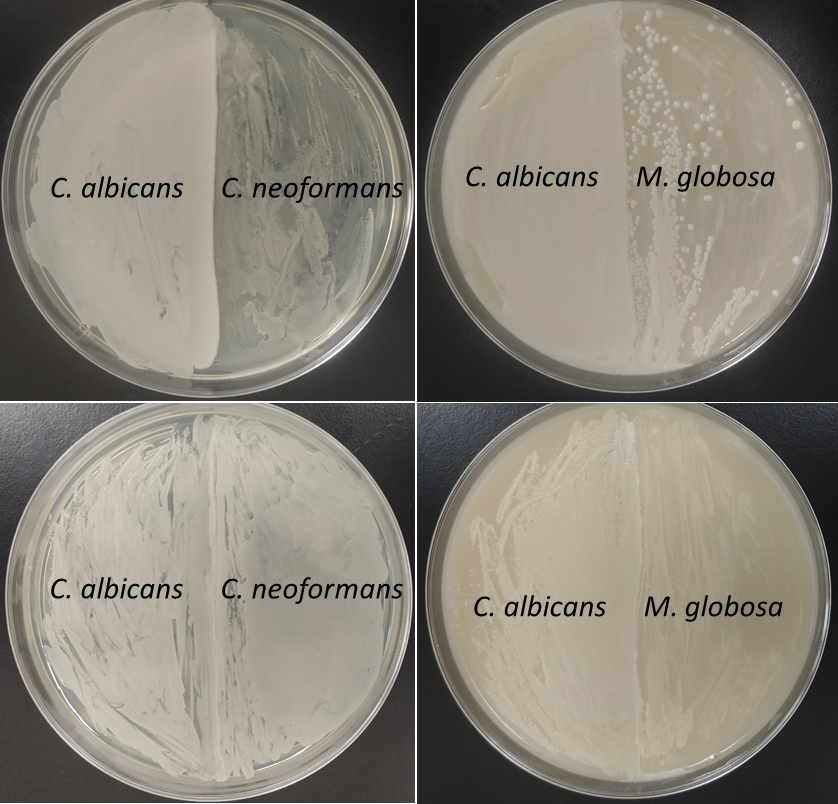

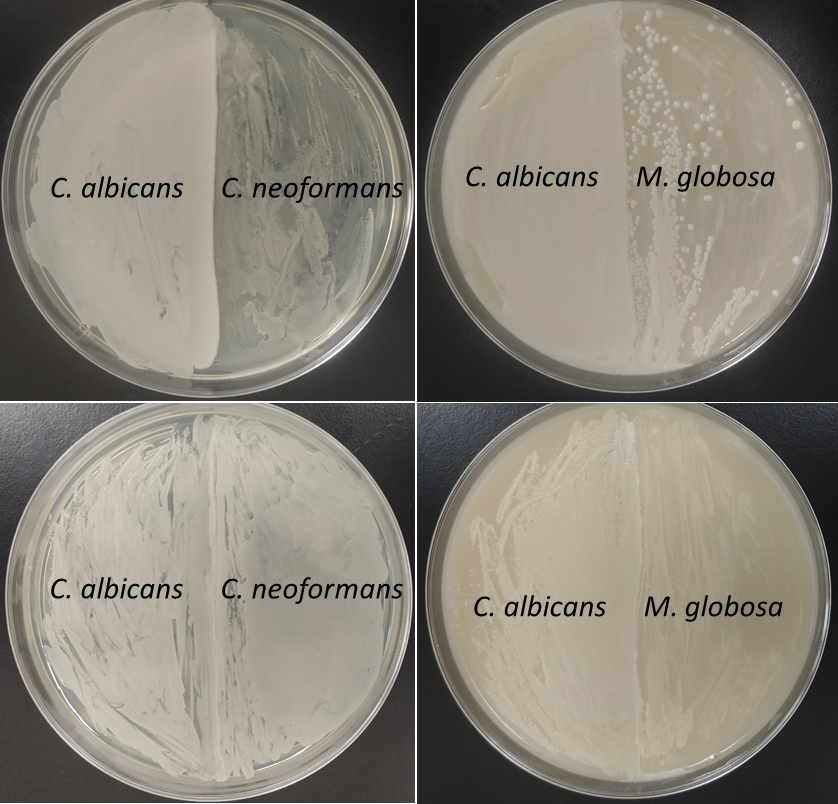

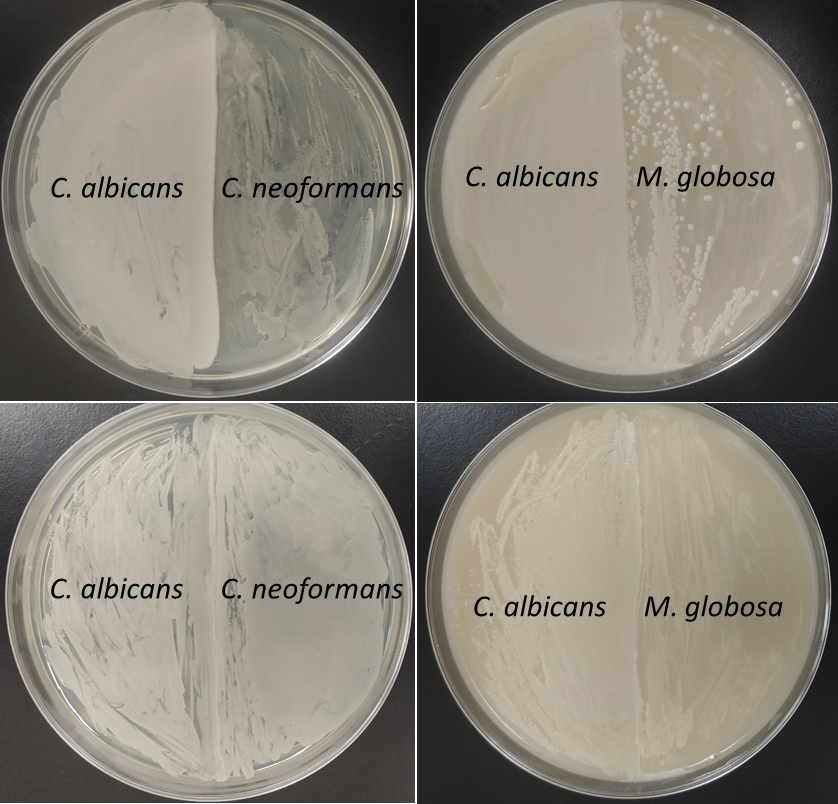

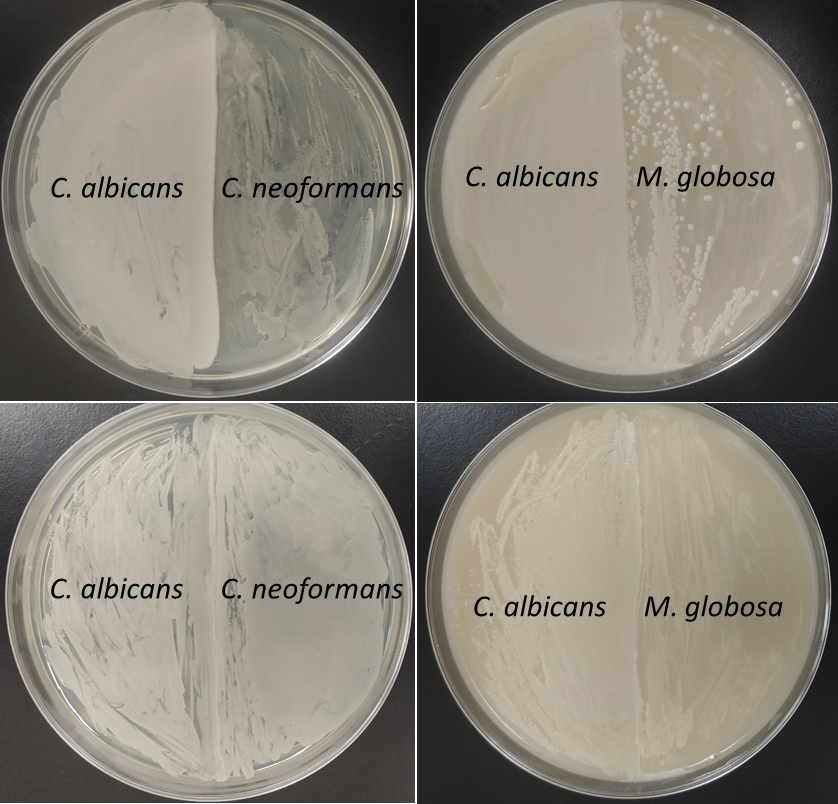

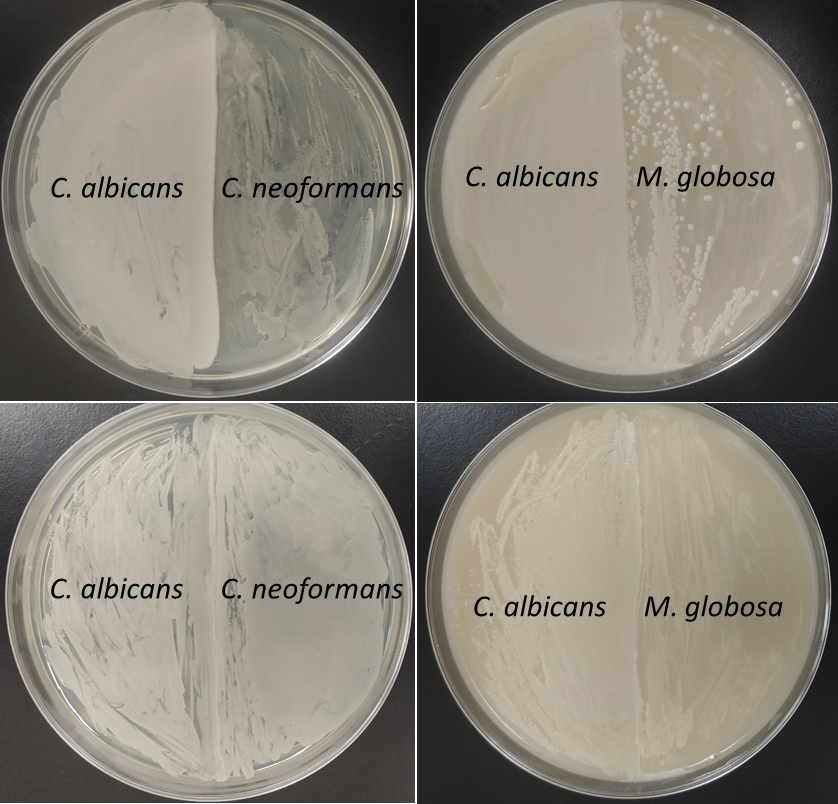

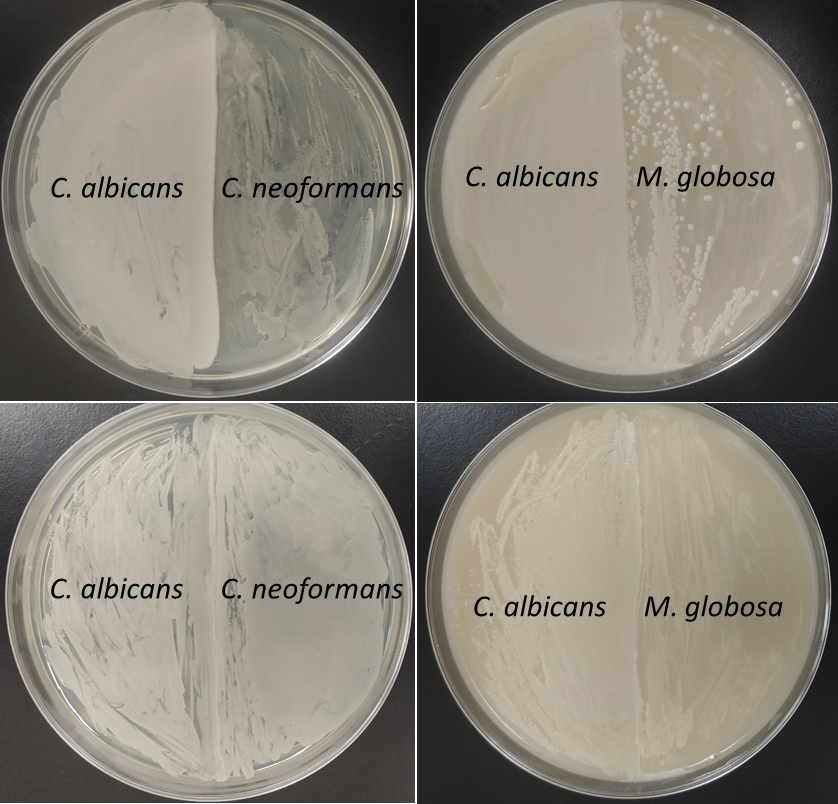

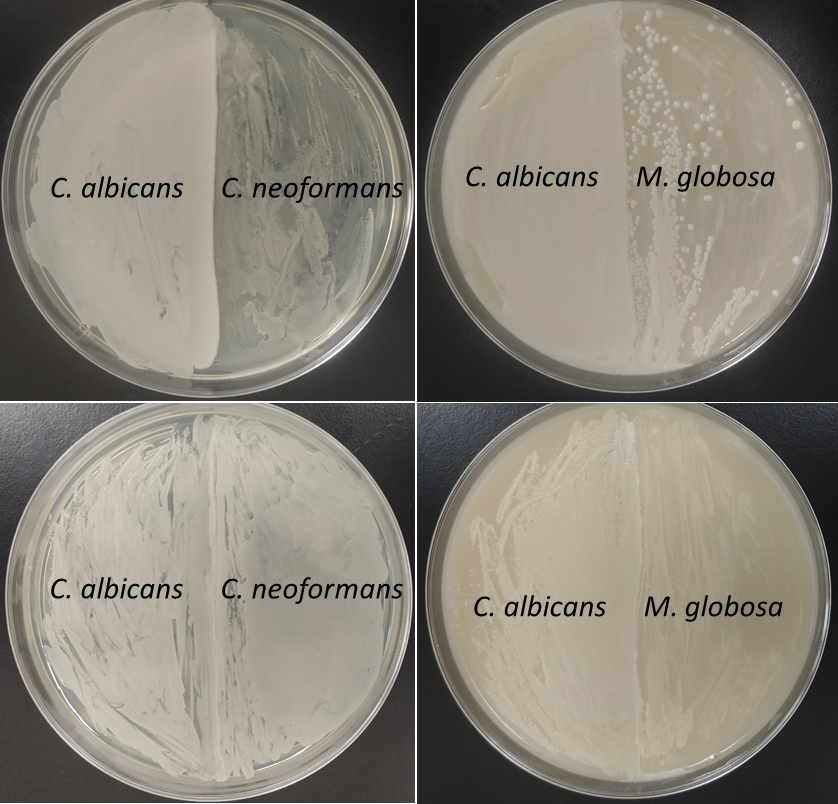

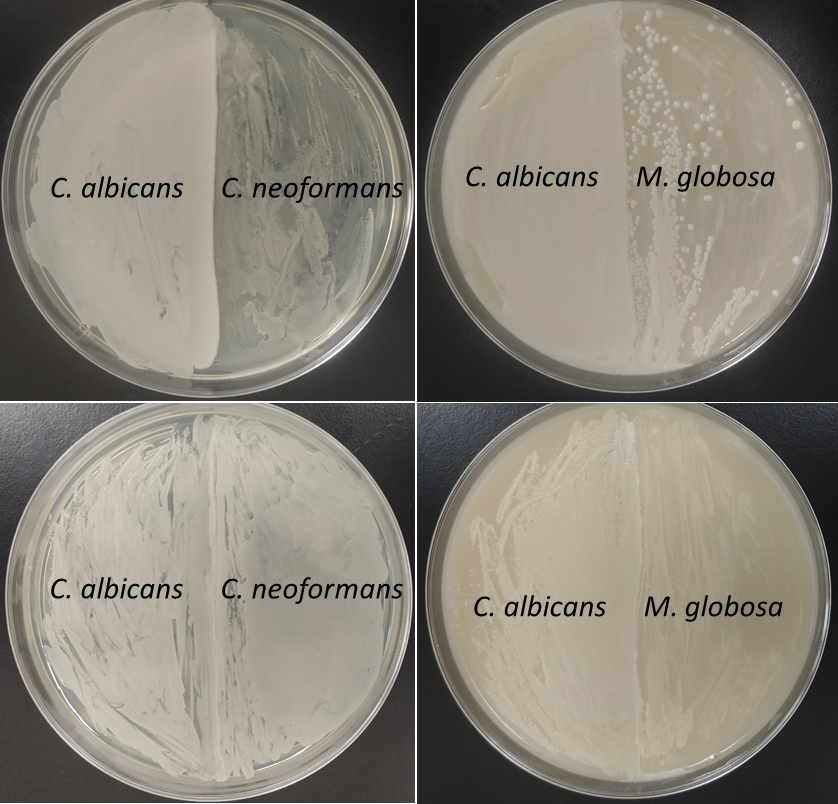
**
